# High level of oxidized nucleosides in thyroid mitochondrial DNA; damaging effects of Fenton reaction substrates

**DOI:** 10.1186/1756-6614-5-24

**Published:** 2012-12-26

**Authors:** Małgorzata Karbownik-Lewińska, Jan Stępniak, Andrzej Lewiński

**Affiliations:** 1 Department of Oncological Endocrinology, Medical University of Łódź, 7/9 Żeligowski St, 90-752, Łódź, Poland; 2 Department of Endocrinology and Metabolic Diseases, Medical University of Łódź, 281/289 Rzgowska St, 93-338, Łódź, Poland; 3 Polish Mother’s Memorial Hospital - Research Institute, 281/289, Rzgowska St, 93-338, Łódź, Poland

**Keywords:** Mitochondrial DNA, Thyroid, Ferrous ion, Hydrogen peroxide, Oxidative damage

## Abstract

**Background:**

The mitochondrial DNA (mtDNA) lies in close proximity to the free radical-producing electron transport chain, thus, it is highly prone to oxidative damage. Oxyphilic type of follicular thyroid carcinoma consists of cells filled – almost exclusively – with aberrant mitochondria. In turn, bivalent iron (Fe^2+^) and hydrogen peroxide (H_2_O_2_) are indispensable for thyroid hormone synthesis, therefore being available in physiological conditions presumably at high concentrations. They participate in Fenton reaction (Fe^2+^+H_2_O_2_→Fe^3+^+^·^OH + OH^-^), resulting in the formation of the most harmful free radical – hydroxyl radical (^·^OH). The same substrates may be used to experimentally induce oxidative damage to macromolecules. The aim of the study was to evaluate the background level of oxidative damage to mtDNA and the damaging effects of Fenton reaction substrates.

**Methods:**

Thyroid mtDNA was incubated in the presence of either H_2_O_2_ [100, 10, 1.0, 0.5, 0.1, 0.001, 0.00001 mM] or FeSO_4_ (Fe^2+^) [300, 150, 30, 15, 3.0, 1.5 μM], or in the presence of those two factors used together, namely, in the presence of Fe^2+^ [30 μM] plus H_2_O_2_ [100, 10, 1.0, 0.5, 0.1, 0.001, 0.00001 mM], or in the presence of H_2_O_2_ [0.5 mM] plus Fe^2+^ [300, 150, 30, 15, 3.0, 1.5 μM]. 8-oxo-7,8-dihydro-2’-deoxyguanosine (8-oxodG) concentration, as the index of DNA damage, was measured by HPLC.

**Results:**

Both Fenton reaction substrates, used separately, increased 8-oxodG level for the highest H_2_O_2_ concentration of 100 mM and in Fe^2+^ concentration-dependent manner [300, 150, and 30 μM].

When Fe^2+^ and H_2_O_2_ were applied together, Fe^2+^ enhanced H_2_O_2_ damaging effect to a higher degree than did H_2_O_2_ on Fe^2+^ effect.

**Conclusions:**

The level of oxidized nucleosides in thyroid mtDNA is relatively high, when compared to nuclear DNA. Both substrates of Fenton reaction, i.e. ferrous ion and hydrogen peroxide, increase oxidative damage to mtDNA, with stronger damaging effect exerted by iron. High level of oxidative damage to mtDNA suggests its possible contribution to malignant transformation of thyroid oncocytic cells, which are known to be especially abundant in mitochondria, the latter characterized by molecular and enzymatic abnormalities.

## Background

Reactive oxygen species (ROS) are generated in animal cells as natural by-products of oxygen metabolism. They participate in numerous important life processes, like cell signaling or host defense against pathogens [1,2]. On the other hand, due to highly reactive nature, ROS are potentially very toxic and they can damage macromolecules, such as DNA, proteins and lipids. Normally, the cell is able to maintain an adequate balance between the formation and removal of ROS. However, when the levels of ROS increase, this balance may be disturbed, leading to oxidative stress, a condition involved in the pathogenesis of many diseases [1,3-5].

The most basic reaction of oxidative stress is Fenton reaction:

(1) $$Fe^{2+}+H_{2}O_{2}\to Fe^{3+}{+}^{\cdot }\mathrm{OH}+OH^{-}$$

Hydroxyl radical (^●^OH) – being the most harmful free radical – is produced during the above reaction. Both substrates of Fenton reaction are normally present in thyroid cells and possess important physiological roles.

H_2_O_2_ is an indispensable factor in the process of thyroid hormone synthesis, acting as an electron acceptor at each step of this process, namely, at iodide oxidation, next, at its organification, as well as at iodotyrosine coupling reactions [6]. H_2_O_2_ is synthesized in the thyroid gland by certain enzymes of a dual oxidase/NADPH oxidase family (NOX/DUOX), with the most convincing experimental evidence found for DUOX2, acting mainly at the apical membrane or extracelluary – in the colloid [7] and for NOX4, acting intracellulary [8]. H_2_O_2_ availability is the rate-limiting step in thyroid hormone biosynthesis, although H_2_O_2_ is produced in large excess compared to the amount of iodide incorporated into proteins. This may be due to relatively high Michaelis-Menten constant of thyroperoxidase (TPO) for H_2_O_2_, which means that relatively high concentrations of H_2_O_2_, as a substrate, are required to properly activate the enzyme [9,10]. It should be stressed that the stimulated thyroid cell generates as much H_2_O_2_ as an activated leukocyte [11]. Large quantities and membrane permeable nature of H_2_O_2_ can lead to its diffusion from the luminal side of the apical membrane back to the cell, potentially creating conditions for a huge oxidative stress.

Iron is an essential element for normal metabolic processes, being a cofactor for many biological reactions. On the other hand, free ionic iron, as a potent generator of ROS, can enhance oxidative stress. In the thyroid gland, iron is bound to TPO and it is required for its biological activity. Activated TPO (hemoprotein) constitutes only approximately 2% of total TPO; it is located in the apical membrane and it exposes its heme-linked catalytic site facing the thyroid follicular lumen [12,13]. This kind of iron compartmentalization constitutes, in a certain sense, defense mechanisms against oxidative damage in the thyroid, caused by heme iron.

Bivalent iron (ferrous ion; Fe^2+^) and/or H_2_O_2_ which – when used together – initiate Fenton reaction, are frequently used to experimentally induce oxidative damage to macromolecules [6,14-22]. Thus, the present study is the next approach to evaluate oxidative damage to macromolecules caused by Fenton reaction substrates but the first one, in which thyroid mtDNA has been used.

Mitochondria remain the main source of ROS, even in thyroid tissue. Furthermore, they are the only cellular organelles in cells that contain their own DNA. This mitochondrial DNA (mtDNA) lies in close proximity to the free radical-producing electron transport chain and it is not protected by histones and polyamines, thus it is highly prone to oxidative damage. Additionally, lack of buffering structures, such as introns, also renders mtDNA more prone to mutations [23]. Consistently, it has been reported that mtDNA is characterized by a higher level of oxidative DNA damage than nuclear DNA [24]. In human mtDNA, over 150 pathogenic mutations have been identified; there is evidence that these mutations lead to a wide variety of degenerative diseases, preferentially in tissues with high energy demands, such as the central nervous system, heart, skeletal muscles and endocrine system [25]. Accumulation of somatic mutations in mtDNA causes deficiencies in oxidative phosphorylation and the electron transport chain, which, in turn, cause both further increased production of ROS and their leakage into the cytoplasm.

The aim of the study was to evaluate the background level of oxidative damage to thyroid mtDNA and the damaging effects of Fenton reaction substrates. Since 8-oxo-7,8-dihydro-2’-deoxyguanosine (8-oxodG) is a major product of oxidatively damaged DNA, this oxidized nucleoside has been used to evaluate oxidative damage to mtDNA. It should be stressed that the level of 8-oxodG, resulting from oxidative mtDNA damage, has never been examined in the thyroid gland under any conditions.

## Methods

### Ethical approval

The procedures, used in the study, were approved by the Ethics Committee of the Medical University of Lodz, Poland.

### Chemicals

Ferrous sulfate (FeSO_4_), hydrogen peroxide (H_2_O_2_), alkaline phosphatase and nuclease P_1_ were purchased from Sigma (St. Louis, MO). MilliQ-purified H_2_O was used for preparing all solutions. All the used chemicals were of analytical grade and came from commercial sources.

### Animals

Porcine thyroids were collected from sixty three (63) animals at a slaughter-house, frozen on solid CO_2_, and stored at −80°C until assay. Three independent experiments were performed. Therefore, three tissue pools were prepared, with twenty one (21) thyroid glands used for each experiment.

### Mitochondrial DNA isolation

Mitochondrial DNA was isolated using an alkaline lysis method [26]. Thyroid tissue was homogenized in chilled homogenization buffer (0.25 M sucrose, 10 mM EDTA, 30 mM Tris–HCl, pH 7.5) and centrifuged at 1000 × g for 3 min at 4°C in order to pellet the nuclei and cellular debris. The supernatant was centrifuged again at 12,000 × g for 10 min at 4°C, to obtain pellet of mitochondria. This pellet was resuspended in 10 mM Tris-EDTA buffer (containing 0.15 M NaCl and 10 mM EDTA, pH 8.0) and then two volumes of freshly prepared 0.18 M NaOH, containing 1% SDS, were added. After 5 min incubation on ice, solution of ice-cold potassium acetate (3 M potassium and 5 M acetate) was added. After another 5 min incubation on ice, mixture was centrifuged at 12,000 × g for 5 min at 4°C. The obtained supernatant was mixed with an equal volume of phenol/chloroform/isoamyl-alcohol (25:24:1) mixture. After centrifugation at 12,000 × g for 5 min at room temperature, mtDNA was precipitated by the addition of five volumes of ethanol (−20°C).

### Mitochondrial DNA incubation

Mitochondrial DNA was incubated in 10 mM potassium phosphate buffer (pH 7.4) at a final volume of 0.5 ml in the presence of either H_2_O_2_ [100, 10, 1.0, 0.5, 0.1, 0.001, 0.00001 or 0.0 mM] or FeSO_4_ [300, 150, 30, 15, 3.0, 1.5, or 0.0 μM] or in the presence of those two agents used together, namely FeSO_4_ [30 μM] + H_2_O_2_ [100, 10, 1.0, 0.5, 0.1, 0.001, 0.00001 or 0.0 mM] or H_2_O_2_ [0.5 mM] + FeSO_4_ [300, 150, 30, 15, 3.0, 1.5, or 0.0 μM]. The reaction was carried out at 37°C for 1 hr in a water bath. Three independent experiments were performed, and in each experiment mtDNA was isolated from twenty one (21) different thyroid glands.

### Evaluation of the 8-oxo-7,8-dihydro-2’deoxyguanosine/2’-deoxyguanosine (8-oxodG/dG) ratio

After incubation, 50 μl of sodium acetate (3 M, pH 5.0) and two volumes of ethanol (20°C) were added to each sample to terminate the reaction. DNA was precipitated by centrifugation (13,000 × g, 5 min); DNA was washed once with 70% ethanol. Thereafter, the DNA sample was dried and dissolved in 20 mM sodium acetate (pH 5.0); the samples were denatured by heating at 95°C for 10 min and, then, cooled on ice for 5 min. The DNA samples were digested to nucleotides by incubation with 8 units of nuclease P_1_ at 37°C for 30 min. Next, pH was adjusted by adding 20 μl of 1 M Tris–HCl and the samples were treated with 4 units of alkaline phosphatase at 37°C for 1 hr. The resulting deoxynucleoside mixture was filtered through a Millipore filter (0.22 mm) and analyzed by HPLC with electrochemical (EC) detection. The HPLC system consisted of a Smartline Pump 1000, Smartline Autosampler 3800, 250 mm × 4 mm Eurosphere-100 C18 column and electrochemical detector EC3000 with measurement cell model Sputnik. An eluent (10% aqueous methanol containing 12.5 mM citric acid, 25 mM sodium acetate, 30 mM sodium hydroxide and 10 mM acetic acid) was used at a flow rate of 1 ml/min. The quantities of 8-oxo-7,8-dihydro-2’-deoxyguanosine (8-oxodG) and of 2’-deoxyguanosine (dG) were measured using two oxidative potentials (600 mV, 900 mV, respectively). The results are expressed as the ratio of 8-oxodG to dG × 10^5^.

### Statistical analyses

Results represent means ± SE. Data were statistically analyzed, using a one-way analysis at variance (ANOVA), followed by the Student-Neuman-Keuls’ test. The level of p < 0.05 was accepted as statistically significant.

## Results

**Figure 1:**
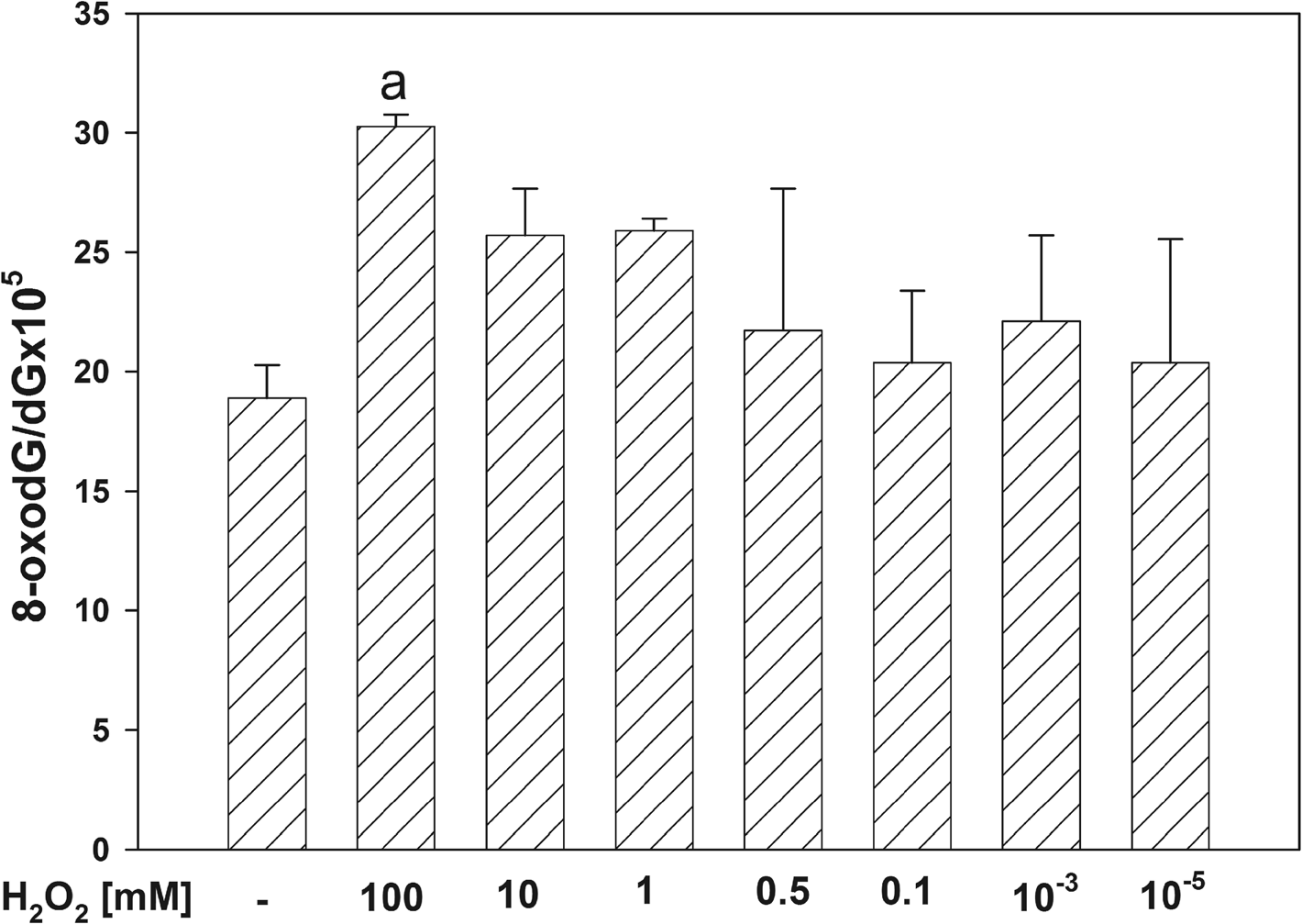
**Oxidative damage to mitochondrial DNA in porcine thyroid.** mtDNA was incubated in the presence of H_2_O_2_ alone [100, 10, 1.0, 0.5, 0.1, 0.001, 0.00001 mM]. Data are expressed as the ratio 8-oxodG/dGx10^5^. Data are from three independent experiments. Values are expressed as mean ± SE (error bars). ^a^p = 0.05 *vs*. control (in the absence of H_2_O_2_).

**Figure 2:**
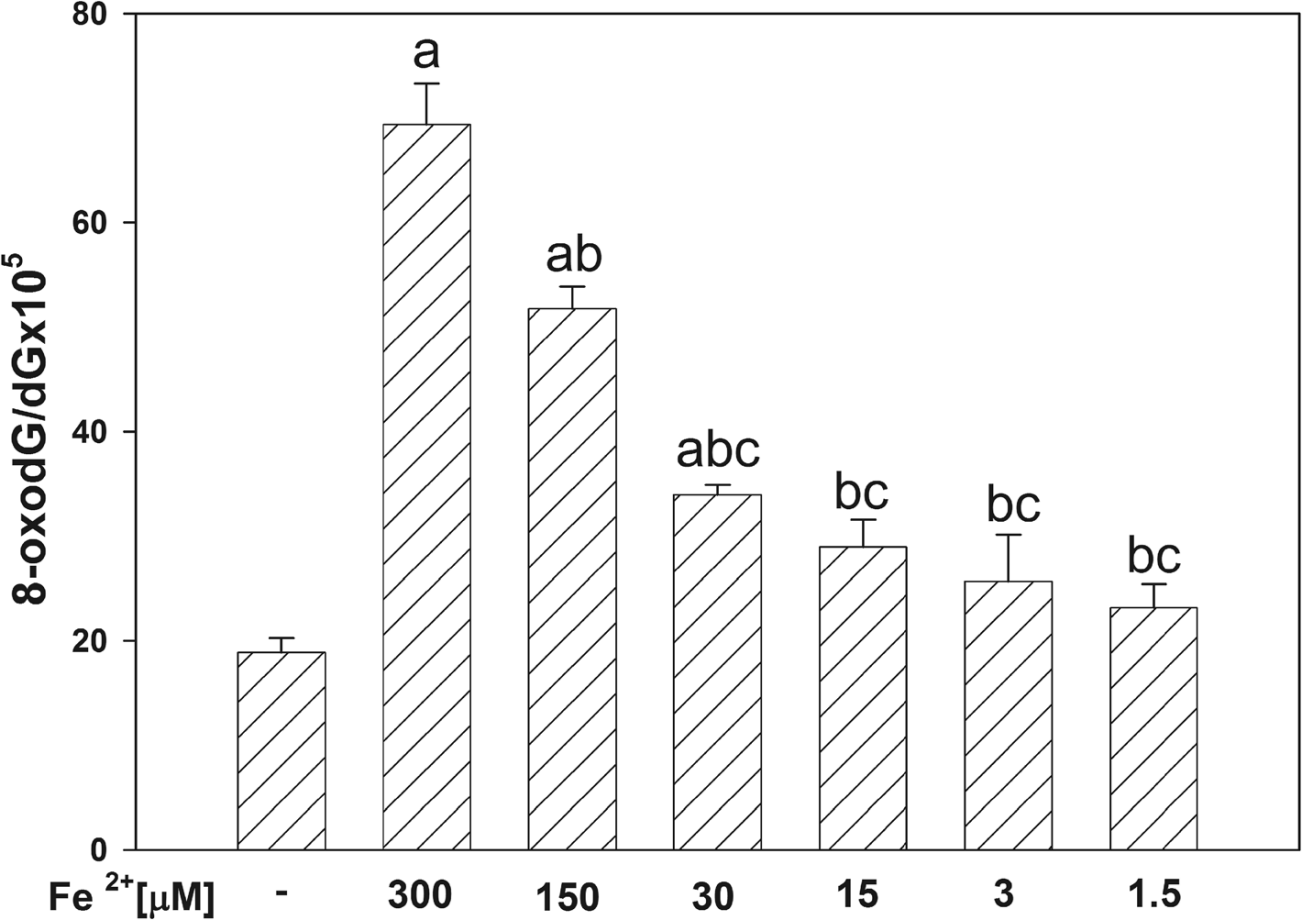
**Oxidative damage to mitochondrial DNA in porcine thyroid.** mtDNA was incubated in the presence of FeSO_4_ (Fe^2+^) alone [300, 150, 30, 15, 3.0, 1.5 μM]. Data are expressed as the ratio 8-oxodG/dGx10^5^. Data are from three independent experiments. Values are expressed as mean ± SE (error bars). ^a^p = 0.05 *vs*. control (in the absence of Fe^2+^), ^b^p = 0.05 *vs*. 300 μM, ^c^p = 0.05 *vs*. 150 μM.

The incubation of mtDNA in the presence of either H_2_O_2_ (Figure 1) or ferrous ions (Figure 2) increased the level of oxidative damage, namely the level of 8-oxodG increased significantly when H_2_O_2_ was used in the highest concentration of 100 mM (Figure 1), and Fe^2+^ increased 8-oxodG level in concentration-dependent manner (for concentrations of 300, 150, 30 μM) (Figure 2).

**Figure 3:**
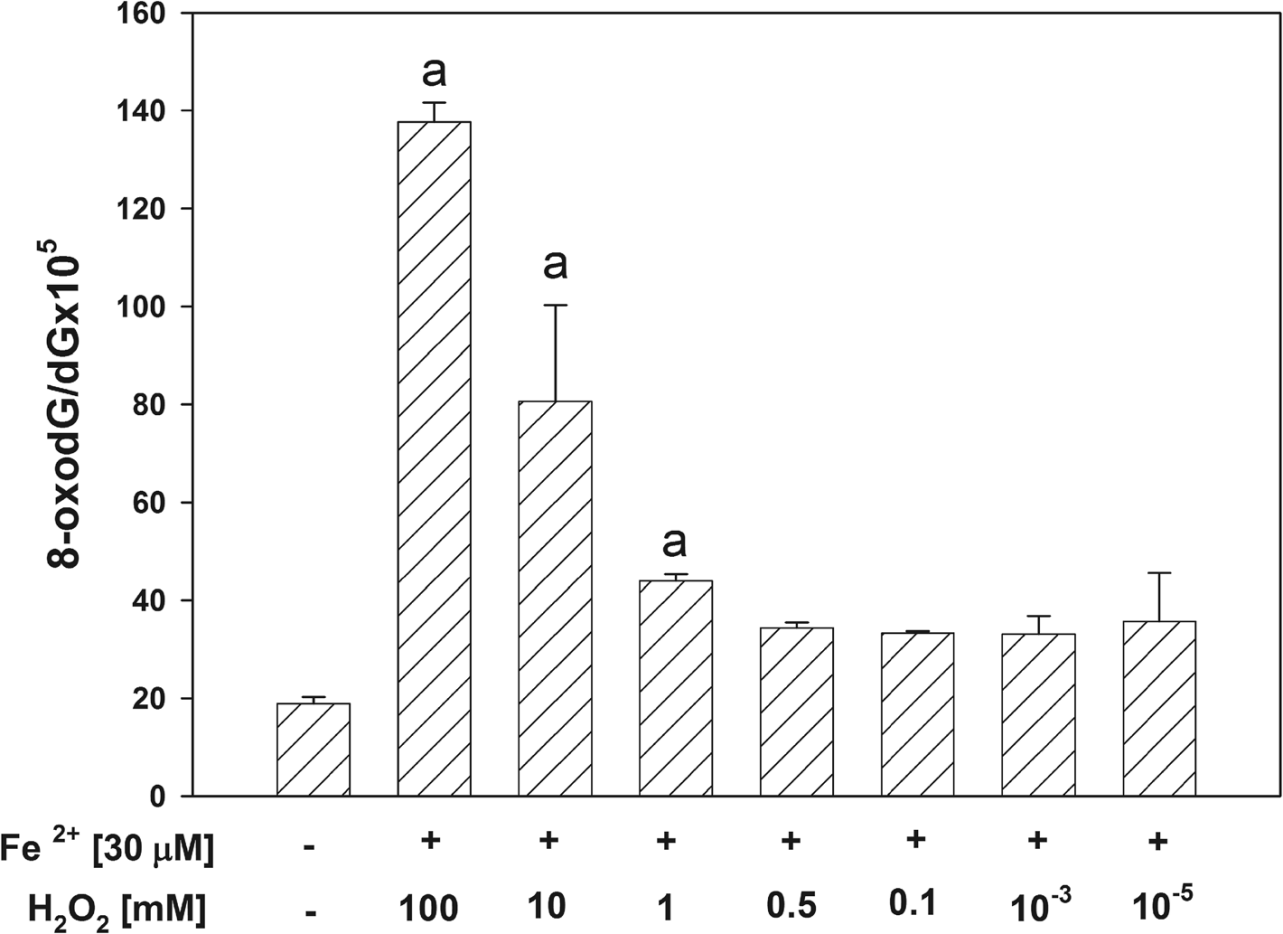
**Oxidative damage to mitochondrial DNA in porcine thyroid.** mtDNA was incubated in the presence of both substrates used together, namely in the presence of 30 μM FeSO_4_ plus H_2_O_2_ [100, 10, 1.0, 0.5, 0.1, 0.001, 0.00001 mM]. Data are expressed as the ratio 8-oxodG/dGx10^5^. Data are from three independent experiments. Values are expressed as mean ± SE (error bars). ^a^p = 0.05 *vs*. control (in the absence of both Fe^2+^ and H_2_O_2_).

When Fe^2+^ [30 μM] was used together with different concentrations of H_2_O_2_, 8-oxodG level in mtDNA increased significantly in H_2_O_2_ concentration-dependent manner (Figure 3). When comparing Figures 1 and 3, the damaging effect of both substrates used together has been much more stronger comparing to damaging effect of H_2_O_2_ alone. First, the range of H_2_O_2_ concentrations [100 mM, 10 mM and 1 mM], which have increased 8-oxodG level, is wider in the presence of Fe^2+^. Second, 8-oxodG level is approximately 4 × higher in the presence of H_2_O_2_ [100 mM] plus Fe^2+^ when compared to the effect of H_2_O_2_ [100 mM] alone. This suggests that the addition of Fe^2+^ strongly enhanced the effect of H_2_O_2_.

**Figure 4:**
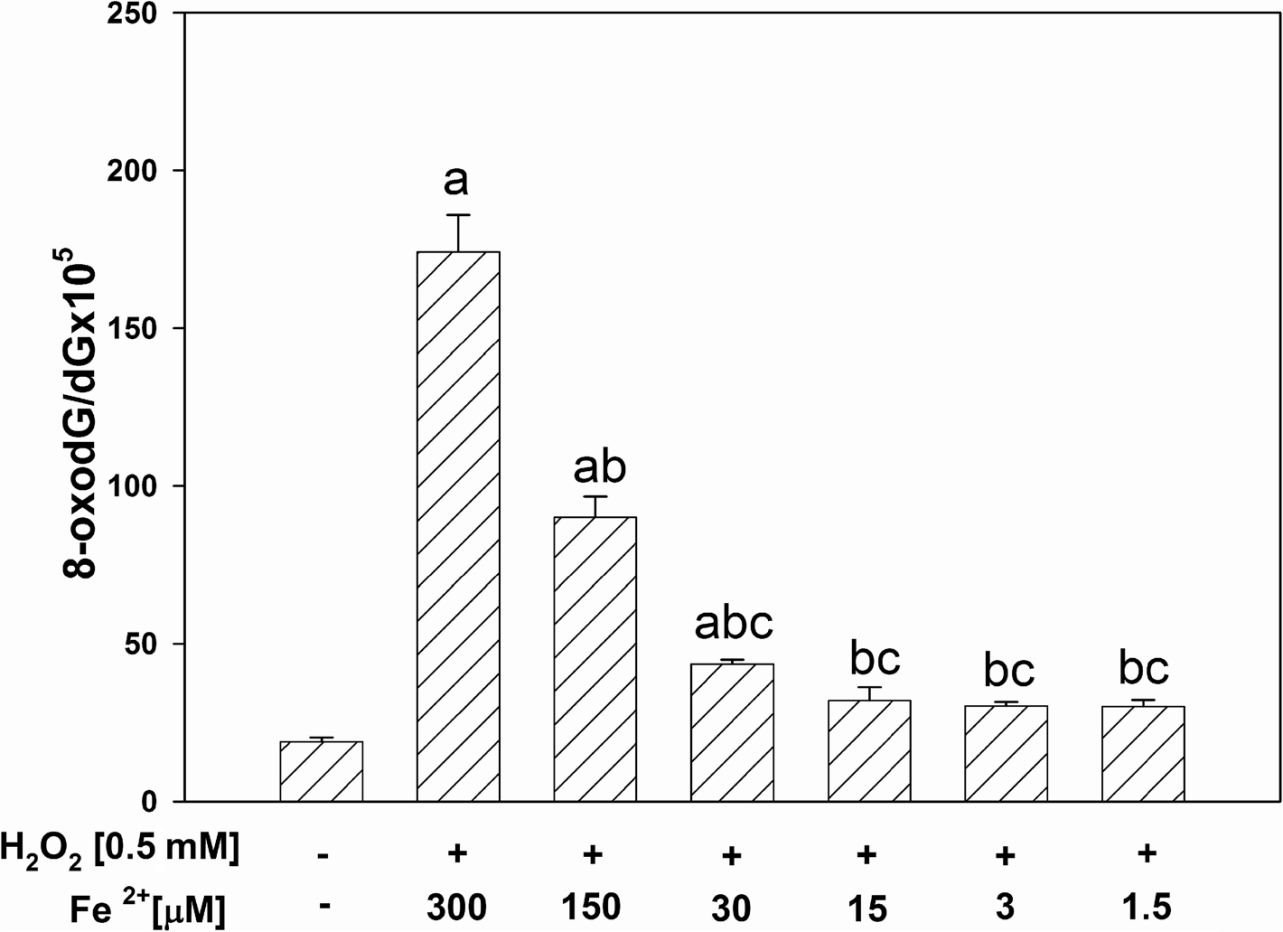
**Oxidative damage to mitochondrial DNA in porcine thyroid.** mtDNA was incubated in the presence of both substrates used together, namely in the presence of 0.5 mM H_2_O_2_ plus FeSO_4_ [300, 150, 30, 15, 3.0, 1.5 μM]. Data are expressed as the ratio 8-oxodG/dGx10^5^. Data are from three independent experiments. Values are expressed as mean ± SE (error bars). ^a^p = 0.05 *vs*. control (in the absence of both Fe^2+^ and H_2_O_2_), ^b^p = 0.05 *vs*. 300 μM, ^c^p = 0.05 *vs*. 150 μM.

In turn, when H_2_O_2_ [0.5 mM] was used together with different concentrations of Fe^2+^, 8-oxodG level in mtDNA increased significantly in Fe^2+^ concentration-dependent manner (Figure 4). These effect was observed for exactly the same concentrations [300, 150 and 30 μM], as when ferrous ion was used separately (compare with Figure 2). However, 8-oxodG level was above 2 × higher in the presence of Fe^2+^ [300 μM] plus H_2_O_2_ [0.5 mM] (Figure 4) than in the presence of Fe^2+^ [300 μM] alone (Figure 2). The difference between damaging effects of Fe^2+^ + H_2_O_2_ and Fe^2+^ alone is not so obvious for two other lower Fe^2+^ concentrations, namely for 150 μM and 30 μM. The results suggest that the addition of H_2_O_2_ enhanced the effect of Fe^2+^.

It should be stressed that Fe^2+^ intensified the damaging effect of H_2_O_2_ stronger than H_2_O_2_ intensified the damaging effect of Fe^2+^.

## Discussion

In mitochondrial and nuclear DNA, 8-oxodG is the most abundant oxidatively damaged product following the exposure to free radicals, and – therefore – it is widely used as a biomarker of oxidative stress and carcinogenesis. According to our knowledge, this study is the first one, in which the level of oxidized nucleosides in mtDNA in the thyroid gland has been measured. The fact that the attempt to measure oxidative damage to mtDNA in the thyroid has not been undertaken before may be due to technical difficulties occurring during mtDNA isolation from the thyroid. In the process of mtDNA isolation significant amount of the mtDNA is lost, especially at the step of isolation of the whole mitochondria; therefore, much more tissue is required and the procedure is more time-consuming, comparing to nuclear DNA isolation.

When designing this study, we expected that physiological damage to mtDNA would be much higher than that one to nuclear DNA. Expectedly, the background oxidative damage to mtDNA in the present study (8-oxodG/dG×10^5^ = from 15.96 to 21.32) was approximately ten (10) times higher than to nuclear DNA, the latter being observed in our earlier study (8-oxodG/dG×10^5^ = from 2.24 to 2.80) [27]. These results are in agreement with data already presented in numerous published studies concerning other tissues. Values for the ratio of mitochondrial to nuclear levels of 8-oxodG range from 2 (in human fibroblasts) [28] to 16 (in rat liver) [24]. That significantly higher background oxidative damage in mtDNA appears to represent evidence for more extensive oxidation of mtDNA comparing to nuclear DNA under physiological conditions. Main reasons for higher sensitivity to oxidative stress of mtDNA, comparing to nuclear DNA, comprise mentioned above such characteristics as proximity of mtDNA to the mitochondrial electron transport chain, being a site of superoxide anion (O_2_^-·^) and H_2_O_2_ generation, as well as the lack of protective histones. It should be also stressed that mtDNA does not possess introns, therefore, the whole mtDNA – when exposed to free radicals – can be damaged.

It should be mentioned that in the last years certain doubts arose concerning the reliability of the measurement of mtDNA oxidation. The question to what extent mitochondrial 8-oxodG levels, measured in experimental conditions, correspond to those typical for physiological conditions (in the living organism) has not been yet adequately answered. Some authors argue, that additional oxidative damage might be induced during the procedure of mitochondria isolation. According to this hypothesis, during the process of mitochondria isolation, organelles may continue to generate oxygen radicals which can contribute to increase *ex vivo* oxidation. Moreover, mitochondria contain large quantities of heme protein with redox-active iron atom, which also may cause extensive oxidation during the isolation [29]. However, it should be underlined that the above hypothesis has not been univocally proved. In turn, even if any additional oxidation occurs during mitochondria isolation or, later on, before 8-oxodG measurement, it is still unknown to what extent such an oxidation contributes to the final result. High ratio of mitochondrial/nuclear 8-oxodG, mentioned above and described for different tissues [29], as well as the value of that ratio equal to 10, as observed by us in the thyroid in the present study, support the statement that oxidative damage to mtDNA is much stronger than that directed against nuclear DNA.

Another aspect which should be discussed in the present study is the sensitivity of thyroid mtDNA to Fenton reaction substrates. In our present study, addition of a single Fenton reaction substrate was sufficient to increase 8-oxodG level in mtDNA. The damaging effects of Fenton reaction substrates, when used separately, suggest that exogenous ferrous ion and exogenous H_2_O_2_ reacted with the other substrate present already (physiologically) in mtDNA. Thus, the exposure to only one of these factors can cause oxidative damage to mtDNA. When both Fenton reaction substrates were applied together, elevations in 8-oxodG levels were, expectedly, higher than those when they were used separately. It should be stressed that ferrous ion revealed stronger damaging effect than H_2_O_2_, both when the substrates were used separately or were applied together. The results on oxidative mtDNA damage suggest that in the thyroid gland iron is a more potent prooxidative factor, when compared to H_2_O_2_. These results are in agreement with our observation for nuclear DNA and also for membrane lipids in our previous study [27]. In turn, as regards the response of nuclear DNA [27] and mtDNA (the present study) to Fenton reaction substrates, mtDNA seems to be less vulnerable. Such results have been expected, as macromolecules with physiologically high oxidative damage are usually less sensitive to additional oxidative abuse. However, the differences in the oxidative response of mtDNA and nuclear DNA are not obvious enough to draw final conclusions as to which of these two kinds of DNA is more sensitive to Fenton reaction substrates.

What is a clinical significance of the present findings is not clear enough. Our results show that excessive amounts of Fe^2+^ or H_2_O_2_ contribute to the increased oxidative damage to mtDNA which, in turn, can lead to mutations, impairment of electron transport chain and loss of mitochondrial functions. Decline of the mitochondria respiratory functions is generally accepted as an important contributor to aging and wide range of degenerative diseases. Dysfunction of mitochondria are also suggested to be a predominant feature in oncocytic tumor transformation. Oncocytic neoplasms are the tumours composed of cells filled – almost exclusively – with mitochondria characterized by molecular and enzymatic abnormalities. They mainly occur in endocrine and exocrine tissues but also have been observed in other organs [23,30]. In the thyroid gland, oncocytic cells (also known as Hürtle cells or oxyphilic cells) are frequently observed in benign and malignant tumors, as well as in chronic inflammatory conditions or in hyperplastic lesions. Hürtle cells are characterized by blocked apoptosis, probably as a consequence of mitochondrial abnormalities. The main reason responsible for oncocytic transformation can be compensatory mechanism, in which the activation of mitochondrial biogenesis pathways constitutes the response to metabolic stress, caused by loss of mitochondrial function. This leads to increase of mitochondrial mass and further intensifies oxidative stress.

Oxidative stress is hypothesized to play a crucial role in thyroid cancer, especially in papillary thyroid carcinoma, initiation [31]. However, the present results allow to propose that oxidative processes substantially contribute to formation of tumors, with oxyphilic type of follicular thyroid carcinoma being of special significance. The frequency of this type of cancer is much lower than one should expect, taking into account huge oxidative damage to mtDNA under normal conditions. It is assumed that – due to enormous oxidative stress in mitochondria – defense mechanisms are perfectly developed in these organelles under physiological conditions, preventing serious consequences, such as cancer. However, with additional insult, the protective mechanisms may be disrupted and the formation of ROS may be even higher, leading to the initiation of thyroid cancer, composed of cells rich in abnormal mitochondria. Further studies are required to confirm such a hypothesis.

## Conclusions

The level of oxidized nucleosides in thyroid mtDNA is relatively high, when compared to nuclear DNA. Both substrates of Fenton reaction, i.e. ferrous ion and hydrogen peroxide, increase oxidative damage to mtDNA, with stronger damaging effect exerted by iron. High level of oxidative damage to mtDNA suggests its possible contribution to malignant transformation of thyroid oncocytic cells, which are known to be especially abundant in mitochondria, the latter characterized by molecular and enzymatic abnormalities.

## Competing interests

Authors declare that they have no competing interests.

## Authors’ contributions

MK-L designed the study, supervised its conducting and prepared the final version of the manuscript. JS carried out the experiments, performed the statistical evaluation and prepared the draft of the manuscript. AL revised the final version of the manuscript. All authors read and approved the final manuscript.
